# Practical Lessons in Fever Nursing

**Published:** 1888-03

**Authors:** 


**Practical Lessons in Fever Nursing.** By J. C. WILSON, A. M., M. D., author of "A Treatise on Continued Fever," Visiting Physician to the Philadelphia Hospital, etc., etc. Pp. 208. J. B. Lippincott Company, publishers.

We look upon this as the most valuable of a series of text-books on nursing published by this firm. The work is thoroughly scientific, and not so loaded down with technicalities and hypotheses but that any nurse of ordinary education can comprehend all of the instructions given. The book should be in the hands of everyone intending to adopt nursing as a profession, while medical men should read it carefully, lest the nurse should know more about this important subject than the practitioner himself.

